# Supplementary material for: Rapid turnover of life-cycle-related genes in the brown algae
Source: Genome Biol. 2019 Feb 14;20:35. doi: 10.1186/s13059-019-1630-6 (PMC6374913; doi:10.1186/s13059-019-1630-6)
Supplement: Supplementary file 3 — Protein alignments, corresponding Gblocks html file and CDS sequences for the lineage conserved gametophyte-biased genes. (ZIP 2109 kb) [file 13059_2019_1630_MOESM3_ESM.zip › Con_GA_alignments/Ec-21_005530.aa_ali.fasta-gb.htm]

Ec-21\_005530.aa.fasta.trans.aa\_ali.fasta


## Gblocks 0.91b Results

Processed file: **Ec-21\_005530.aa.fasta.trans.aa\_ali.fasta**  
Number of sequences: **2**  
Alignment assumed to be: **Protein**  
New number of positions: **903** (selected positions are underlined in blue)

```
                         10        20        30        40        50        60
                 =========+=========+=========+=========+=========+=========+
Ec-21_005530     MAFRRVRRRYIDARKNSNIKSIVEELLGAIEGNGMTIRTWFEIMDQLSNNNMINQSEFSK
MSTRG.11165.1    ------------------------------------------------------------
                                                                             


                         70        80        90       100       110       120
                 =========+=========+=========+=========+=========+=========+
Ec-21_005530     GMEMLQNHSTERMKQPVLSAEQIQELFVYMDANHDGILVLNEVALACKVEHYKTTSSQEM
MSTRG.11165.1    ------------------------------------------------------------
                                                                             


                        130       140       150       160       170       180
                 =========+=========+=========+=========+=========+=========+
Ec-21_005530     DAITSMMQALDDYMGVHYMKIRDLFVHLDRDKSDGLSRAEIRCGFHAIQAEAKRKQEAAV
MSTRG.11165.1    ------------------------------------------------------------
                                                                             


                        190       200       210       220       230       240
                 =========+=========+=========+=========+=========+=========+
Ec-21_005530     RRKQKEAVKRAIREDEARIAEAKREEMDRLVYKQRRLSMAGEAAWILAPLTSQHDPWVTE
MSTRG.11165.1    ------------------------------------------------------------
                                                                             


                        250       260       270       280       290       300
                 =========+=========+=========+=========+=========+=========+
Ec-21_005530     ENGLLYKAARGRAPAQALIVYFRSYKPDDGPVYGVIMHPREFLGNVTMRVTLLFKYNDDK
MSTRG.11165.1    ------------------------------------------------------------
                                                                             


                        310       320       330       340       350       360
                 =========+=========+=========+=========+=========+=========+
Ec-21_005530     YAGPARILHPTGGAFEAANASSAAFVSKPELRYIETDSEWRDVIGRQFASKSEARLHHIS
MSTRG.11165.1    ------------------------------------------------------------
                                                                             


                        370       380       390       400       410       420
                 =========+=========+=========+=========+=========+=========+
Ec-21_005530     KWIRDQLATLSAAEEKEPRVSASAVKESLGEVMEFVNNPDNLWAVTREMLETIVSYIRAP
MSTRG.11165.1    ------------------------------------------------------------
                                                                             


                        430       440       450       460       470       480
                 =========+=========+=========+=========+=========+=========+
Ec-21_005530     QSESDEAPVLAATILWKLSEYEDFVLPLLRADAAGKCAAAIEELGPPRAGPLQYWLQVSV
MSTRG.11165.1    ------------------------------------------------------------
                                                                             


                        490       500       510       520       530       540
                 =========+=========+=========+=========+=========+=========+
Ec-21_005530     QTRVTGVLCRIGCAASVDGPVRLRVASALIAVVARPRGPLEARKDYREGADGKILSFASF
MSTRG.11165.1    ------------------------------------------------------------
                                                                             


                        550       560       570       580       590       600
                 =========+=========+=========+=========+=========+=========+
Ec-21_005530     ALGNLVATSTSIRGSIAHRSSLFDALVSALRPATGGAGGARGGRSVGGVLGGSPLQAVAC
MSTRG.11165.1    ------------------------------------------------------------
                                                                             


                        610       620       630       640       650       660
                 =========+=========+=========+=========+=========+=========+
Ec-21_005530     AAFCLAHVTTGESAPLLTRGKILSACLTLSGLLDVCVDQLEAGVGDSSSARGATDGSASE
MSTRG.11165.1    ------------------------------------------------------------
                                                                             


                        670       680       690       700       710       720
                 =========+=========+=========+=========+=========+=========+
Ec-21_005530     VFPITVPNDDAAGAAAATGESGTLSVTMVMESAGKALWGCLSHLSDSTNGDDSTSRSIRD
MSTRG.11165.1    ------------------------------------------------------------
                                                                             


                        730       740       750       760       770       780
                 =========+=========+=========+=========+=========+=========+
Ec-21_005530     SSSCEGSVGSPDRTELSSGGISGGASVDSGCQAPDLKGNEQGALSAAGIPSGNAEGGVSI
MSTRG.11165.1    ------------------------------------------------------------
                                                                             


                        790       800       810       820       830       840
                 =========+=========+=========+=========+=========+=========+
Ec-21_005530     CGGDDSITDRPSMSPRHILSKCALTLLGLLSQVAKHHAHRRSSVGVESTGNDPNCKRMSR
MSTRG.11165.1    ------------------------------------------------------------
                                                                             


                        850       860       870       880       890       900
                 =========+=========+=========+=========+=========+=========+
Ec-21_005530     SGRTGSTSSTKVSSNIDHSETTGKSNSNGGNSSIGGVPHSIVAIAGSCLSRVAQDIDLAA
MSTRG.11165.1    ------------------------------------------------------------
                                                                             


                        910       920       930       940       950       960
                 =========+=========+=========+=========+=========+=========+
Ec-21_005530     GPPGAMESVLALLLPSAGLLVRQQAALTLSAIAGQGFQTLSDFRTEKAAVAAAARWSCGG
MSTRG.11165.1    ------------------------------------------------------------
                                                                             


                        970       980       990      1000      1010      1020
                 =========+=========+=========+=========+=========+=========+
Ec-21_005530     GGNDSERNGGSDDAAQWPGQGEGGRERKRLLLWGTFCAGVVEGTGAQIILDCALPGPSSR
MSTRG.11165.1    ------------------------------------------------------------
                                                                             


                       1030      1040      1050      1060      1070      1080
                 =========+=========+=========+=========+=========+=========+
Ec-21_005530     PNSRLLRERAALAFSLVCLCAGLRPASSAVQGGIVSLLRCRDGEVVGGAATAALWALCRV
MSTRG.11165.1    ------------------------------------------------------------
                                                                             


                       1090      1100      1110      1120      1130      1140
                 =========+=========+=========+=========+=========+=========+
Ec-21_005530     PVNRKAFAKTRCFDYLLLTSITRSRALVCCTLSENEGVVTSFCSKRLQKRQIYPISLLVE
MSTRG.11165.1    ------------------------------------------------------------
                                                                             


                       1150      1160      1170      1180      1190      1200
                 =========+=========+=========+=========+=========+=========+
Ec-21_005530     PENALQITNHGGSIAKLVSWATPLNGDHNSEDLSRMITVEDNAKTASCLGVIRRCLLLPE
MSTRG.11165.1    ------------------------------------------------------------
                                                                             


                       1210      1220      1230      1240      1250      1260
                 =========+=========+=========+=========+=========+=========+
Ec-21_005530     GMKALADGLASYSLIQLVLQLLRVGVWVKSVLPLTLREAQGVLFAVQDRAEYRGYCESIC
MSTRG.11165.1    ------------------------------------------------------------
                                                                             


                       1270      1280      1290      1300      1310      1320
                 =========+=========+=========+=========+=========+=========+
Ec-21_005530     GGDPFGVEDTLALQLQR-----PGVKYAALAGDAVVGLASLSVTLASRRRLAVNGTVESV
MSTRG.11165.1    ---XFSVEDTLALQLKRHVEATPGAQYADLVGDAVVGLASLSMTLVSRRRLAVNGTLESI
                     #############     ######################################


                       1330      1340      1350      1360      1370      1380
                 =========+=========+=========+=========+=========+=========+
Ec-21_005530     DRLLARSMAIESGGG---REKGSKGDHEAVRNMAKLLKNLSSVPEAHRRLSNRPLLPLIT
MSTRG.11165.1    GKLLARTMSFESGAGNGDKRKGRKGRREAIRNLMKLLKNLSSVPEAHQRLSGRPLLLFIN
                 ###############     ########################################


                       1390      1400      1410      1420      1430      1440
                 =========+=========+=========+=========+=========+=========+
Ec-21_005530     RLWEESPDEATSQLACHVLANLSRSDVPGVRNRLFQLHLAAES-EVVRRTRASGGAGTTP
MSTRG.11165.1    RLWEESPDEITSKLACHVLANLSRSDVPGVRNRLFQSHLAAESTRMIRGTSVAIDGGSSS
                 ###########################################    #############


                       1450      1460      1470      1480      1490      1500
                 =========+=========+=========+=========+=========+=========+
Ec-21_005530     APIAKQKPSAAVPRQ---GFAASSTATRAQHGETNSRDDDTVSLSSSVFRRDGFVEGHRY
MSTRG.11165.1    GPAPPEQLSTTVHQKQPRDLIATGAATGAHFGSNDS-DDESLSISSSLFRRDRVDKRDRL
                 ############         ############### #######################


                       1510      1520      1530      1540      1550      1560
                 =========+=========+=========+=========+=========+=========+
Ec-21_005530     KGNGCTSDAKSAAVLLSQEALRRDARRDAQYRLRLMPAALWGSRARHVDDGCSGGEDGLD
MSTRG.11165.1    NG---VGIAHSASVASSEGMARRDARLDAQYRFRLMPAALWGDRASNFSAAGEGNK-GDE
                 ##      ##############################################      


                       1570      1580      1590      1600      1610      1620
                 =========+=========+=========+=========+=========+=========+
Ec-21_005530     VAGIVEKQRTRQTFLTAGLDEASGAVAVESATVTSTLNARHDR-----HQAKRDGSCPSK
MSTRG.11165.1    EATQIAVHEDQQTFLTAAHRAGTAGMETGSPTTDKRNTSRMKASVKCPGVGERGGGGNKR
                                              ###########                    


                       1630      1640      1650      1660      1670      1680
                 =========+=========+=========+=========+=========+=========+
Ec-21_005530     QKIGGAAAVVVQP-SVGDFDDGGGDGIVFQGTAKVSQAASLKRPAVTANVPLRWQSRVVA
MSTRG.11165.1    AVVAVQEPSSVHPGGVREFITSAG-EVALPTGATSPQAASMKRPAVTSSVPLRWQSRVVA
                                                 ############################


                       1690      1700      1710      1720      1730      1740
                 =========+=========+=========+=========+=========+=========+
Ec-21_005530     LSSLRDSLNGG-ATRGSPSMGQRMAKARCRARQRSKENRQHEFTSTTACKDMNKGNNSNE
MSTRG.11165.1    LSSLRDTFTSGTTVRYSPSMGRRMARTRCRAYQCNKERRSEANNPAFSLREEVTMAAGHS
                 ###########   #########################                     


                       1750      1760      1770      1780      1790      1800
                 =========+=========+=========+=========+=========+=========+
Ec-21_005530     TQESKEVTSSDSASPDQRCR-------PSKI----GDTRMTLAEWTSR-------EDVHV
MSTRG.11165.1    RTAPIATATCDNKDTAETCGNQDDADEAYKITPAESDRRTSVTNWTSRGVGTALRGDTRG
                                                     ############            


                       1810      1820      1830      1840      1850      1860
                 =========+=========+=========+=========+=========+=========+
Ec-21_005530     PGGEK--------STDDSGGCGRGAEGILSLVEGGRPSTAPMHSDGKVIMRPLGLGQVDN
MSTRG.11165.1    ISGSVGPDESSNFKNKDGHGDGKGAGGVFSLAEGTRPSTSLTNTGRKAMARPSGPGSGDV
                                 ############################################


                       1870      1880      1890      1900      1910      1920
                 =========+=========+=========+=========+=========+=========+
Ec-21_005530     GVPGGSLEPPSVVFEPGSSRLRTRRIILDRVYRFTRHQEQS-----------------TI
MSTRG.11165.1    DVGGGEIEPPSVVFEAGFSALRRRRTILDRIYRFTRYQGHNGVSTGCKKGVQEPRPIRSI
                 ######################################                      


                       1930      1940      1950      1960      1970      1980
                 =========+=========+=========+=========+=========+=========+
Ec-21_005530     MGAPSGSTDTNRGTTEFNKGSAPDPAPQAGLTSSGS------------------------
MSTRG.11165.1    RQATSNPPEPESSSANIEAGASPISRESTKRGENSTHESVVSNQRKRYPNDLQPWKPPML
                                                                             


                       1990      2000      2010      2020      2030      2040
                 =========+=========+=========+=========+=========+=========+
Ec-21_005530     ---PEDPGKAGGHFSKETIILEPTSPTTHFVFKPPPRNCVDSEGRVVLEAS-SEGVRIKD
MSTRG.11165.1    FQFSKEPLKVGRASNEETVVLDPTSPATHFVFKPPPHSYVDGQGRVVVEAASSEGLAGKG
                       ############################################  ########


                       2050      2060      2070      2080      2090      2100
                 =========+=========+=========+=========+=========+=========+
Ec-21_005530     DDRIRLDRFQHVPGCMVCRGSLGHVKGMDGRYYHHYASSDRMWYMPEHPGPYPTPAEPPS
MSTRG.11165.1    DDRMRLDRFQHVPGCLVCLGSVGHVKGMDGRLYHHYTSSDRMRYMPEHPGPYPPPAEPPS
                 ############################################################


                       2110      2120      2130      2140      2150      2160
                 =========+=========+=========+=========+=========+=========+
Ec-21_005530     LEKLGILPPEPEPPLSPVSGEVVLPSPEDLPGTEPPNAPPLSRHYMKVSTEACSRCQQGG
MSTRG.11165.1    LERLGIQPPKPEPPKSPASGQVVLPFPEELTESDQPNAPPLPRHYMEMSREACSRCHQGG
                 ############################################################


                       2170      2180      2190      2200      2210      2220
                 =========+=========+=========+=========+=========+=========+
Ec-21_005530     KCPGQGLCPVMGVVPVESVILLVEEPRLRDDFDDLGEAVVAEDGRAWSVERSSVFSQRKR
MSTRG.11165.1    KCPGQGACPVMGVVPVESAVLLVDEPRLRDDFDDLGEAVMAESGRAWSVERSSIFSQRKR
                 ############################################################


                       2230      2240      2250      2260      2270      2280
                 =========+=========+=========+=========+=========+=========+
Ec-21_005530     QCESRAVLNTDKVKRSRFEMSFGRASKMQRFPRFVAEGNRDIIIKKTSVKQEIAVIRETL
MSTRG.11165.1    QCESQAVFNTEKVKRKRFETSFGRASKLQRFPRFVAEGNRDIIIKKTSVKQEVALVRETL
                 ############################################################


                       2290      2300      2310      2320      2330      2340
                 =========+=========+=========+=========+=========+=========+
Ec-21_005530     WAHYNTLLSSFNYYVSLDGVFGEPDGAAFQLGRDQYSAFLSDILHTREKENKVVNDELNR
MSTRG.11165.1    WVHYDTLLSSYSYYAALDGVFGEPDSSAYQLGRDQYEAFLADILHAREKENKIVYDEVRK
                 ############################################################


                       2350      2360      2370      2380      2390      2400
                 =========+=========+=========+=========+=========+=========+
Ec-21_005530     TFAKVNAEEDSSTASGLANLDRRLCEQEWMECMVRIAAILYRKETNNGSVARALSRFMAE
MSTRG.11165.1    VFIIVNVEEESSSVAGLANLDRRLCEQEWMECMVRIGAALYRKETNNGSVARALSRFMAE
                 ############################################################


                       2410      2420      2430      2440      2450      2460
                 =========+=========+=========+=========+=========+=========+
Ec-21_005530     LVHRLPKAALLDPDAYRREWLYKEGLEKALTKRIEGFRALFQTYPKNNLAVKAARRRIQV
MSTRG.11165.1    LAQRLPKGALLDADTYRKEKLYKEGVEKVLTRRIEGFRALFQTYPINNLAMKAARRRLQV
                 ############################################################


                       2470      2480      2490      2500      2510      2520
                 =========+=========+=========+=========+=========+=========+
Ec-21_005530     PKEELLRFSLTDWEMLVDDAGIFRMLQRAKVKRVFIQSRMLVTDELVDRTKNCCLTPVDF
MSTRG.11165.1    PK----------------------------------------------------------
                 ##                                                          


                       2530      2540      2550      2560      2570      2580
                 =========+=========+=========+=========+=========+=========+
Ec-21_005530     LEAMVRLADALPRQGDLWPTTEESFEEFCSVLFSGLETRWAGRLRVHMAGSDDDGRTVLN
MSTRG.11165.1    ------------------------------------------------------------
                                                                             


                       2590      2600      2610      2620      2630
                 =========+=========+=========+=========+=========+=
Ec-21_005530     FRNFMKNTGIETREATEDFAASCAIEDDRKSVHSLGADMEECAISEQRSAR
MSTRG.11165.1    ---------------------------------------------EVRGQQ
```

```
Parameters used
Minimum Number Of Sequences For A Conserved Position: 2
Minimum Number Of Sequences For A Flanking Position: 2
Maximum Number Of Contiguous Nonconserved Positions: 8
Minimum Length Of A Block: 10
Allowed Gap Positions: None
Use Similarity Matrices: Yes
```

```
Flank positions of the 14 selected block(s)
Flanks: [1265  1277]  [1283  1335]  [1341  1423]  [1428  1452]  [1462  1476]  [1478  1502]  [1509  1554]  [1590  1600]  [1653  1691]  [1695  1719]  [1777  1788]  [1817  1898]  [1987  2030]  [2033  2462]  

New number of positions in Ec-21_005530.aa.fasta.trans.aa_ali.fasta-gb:  903  (34% of the original 2631 positions)
```
